# Aerobic exercise is associated with alleviation of skeletal muscle atrophy in CT26 tumor-bearing mice: insights from transcriptomic and weighted gene co-expression network analyses

**DOI:** 10.1186/s12891-026-10131-5

**Published:** 2026-06-26

**Authors:** Jiahao Lv, Junwei Wang, Jia Fan, Junzhi Sun, Yang Liu, Shuling Zhang

**Affiliations:** School of Sports Medicine and Health, Chengdu Sport University, Chengdu, China

**Keywords:** Cancer cachexia, Skeletal muscle atrophy, Aerobic exercise, Transcriptome, Weighted gene co-expression network analysis, S100a8, CT26 tumor-bearing mice

## Abstract

**Background:**

Cancer-associated skeletal muscle atrophy is a major manifestation of cachexia and is closely associated with inflammation, metabolic disturbance, and muscle structural deterioration. Aerobic exercise has been considered a promising non-pharmacological intervention, but its molecular mechanisms in tumor-associated muscle wasting remain incompletely understood. This study aimed to investigate transcriptomic changes associated with the effects of aerobic exercise on skeletal muscle wasting-related alterations in CT26 tumor-bearing mice and to identify candidate hub genes using weighted gene co-expression network analysis.

**Methods:**

Eight-week-old specific pathogen-free male BALB/c mice were randomly assigned to four groups: control, exercise, tumor-bearing, and tumor-bearing plus exercise. CT26 cells were subcutaneously inoculated to establish the tumor-bearing model. Mice in the exercise groups underwent treadmill-based aerobic training for 4 weeks. Gastrocnemius muscles were collected for hematoxylin-eosin staining and transcriptome sequencing. Differentially expressed genes were identified, followed by Gene Ontology, Kyoto Encyclopedia of Genes and Genomes, and gene set enrichment analyses. Weighted gene co-expression network analysis was further performed to identify phenotype-related modules and candidate hub genes.

**Results:**

Tumor-bearing mice showed reduced gastrocnemius wet weight and histological abnormalities characterized by disorganized muscle fiber arrangement and structural damage. Compared with the tumor-bearing group, the tumor-bearing plus exercise group exhibited a significant increase in gastrocnemius wet weight alongside a qualitative trend toward histomorphological recovery. Transcriptomic analysis showed that tumor burden upregulated pathways related to inflammation and protein degradation, while downregulating pathways associated with energy metabolism and maintenance of muscle structure. Aerobic exercise was associated with partial reversal of these transcriptional trends. Enrichment analyses indicated that the differentially expressed genes were mainly involved in the PI3K-Akt, NF-kappa B, and IL-17 signaling pathways. Weighted gene co-expression network analysis identified an inflammation-related module closely associated with the tumor-bearing phenotype, in which S100a8 was recognized as a candidate hub gene.

**Conclusions:**

Aerobic exercise was associated with a significant improvement in gastrocnemius wet weight and a mitigating trend in histomorphological abnormalities in CT26 tumor-bearing mice, which may be associated with remodeling of inflammation- and metabolism-related transcriptional networks. S100a8 may represent a candidate hub gene associated with this process, although further functional validation is required.

**Supplementary Information:**

The online version contains supplementary material available at https://doi.org/10.1186/s12891-026-10131-5.

## Background

Skeletal muscle atrophy is one of the common complications of malignant diseases, especially cancer, and is characterized by a marked decline in muscle mass and function, with its incidence gradually increasing as the disease progresses [1]. Cancer-associated cachexia has been reported to occur in up to approximately 70% of patients with cancer, and in patients with advanced disease, skeletal muscle loss can severely impair tolerance to chemotherapy and quality of life [2, 3]. Reports from the National Cancer Institute have indicated that, among patients at the terminal stage, approximately 80% exhibit marked depletion of both muscle and adipose tissue, and this process directly contributes to death in 20%-30% of patients [1, 2]. Relevant epidemiological investigations have also suggested that the decline in muscle mass and function under tumor-bearing conditions has shown an increasing trend year by year, seriously affecting rehabilitation outcomes and increasing the medical burden [3]. Therefore, it is of great importance to identify widely applicable non-pharmacological interventions and to clarify their molecular basis.

Exercise intervention is considered an effective non-pharmacological strategy for alleviating muscle loss. For example, moderate-intensity aerobic exercise can improve mitochondrial function, reduce inflammatory responses, and promote the recovery of muscle metabolism [4]. However, systematic studies on the effects and mechanisms of exercise intervention under tumor-bearing conditions remain limited [5]. The development of transcriptome sequencing and weighted gene co-expression network analysis (WGCNA) has provided new technical approaches for elucidating the molecular mechanisms by which exercise regulates skeletal muscle homeostasis, enabling a deeper understanding of these processes at the gene network level [6, 7].

Based on this, the present study established a CT26 tumor-bearing mouse model and implemented a 4-week aerobic exercise intervention. From a transcriptomic perspective, we analyzed genes and signaling pathways associated with tumor-related skeletal muscle wasting and exercise-induced changes in gastrocnemius muscle. By integrating weighted gene co-expression network analysis (WGCNA), we further explored phenotype-related gene modules and candidate hub genes, with the aim of providing preliminary molecular evidence for the potential role of aerobic exercise in tumor-associated muscle wasting.

## Materials and methods

### Animals and experimental grouping

Forty-eight specific pathogen-free (SPF) male BALB/c mice aged 8 weeks were purchased from SPF (Beijing) Biotechnology Co., Ltd. [Laboratory Animal Production License No. SCXK (Jing) 2019-0010]. All animal housing and experimental procedures were approved by the Ethics Committee of Chengdu Sport University (Approval No. Chengti Ethics [2022] 7). Mice were housed in standard rodent cages under SPF conditions at 21–23 °C, 40–60% relative humidity, and a 12 h light/dark cycle [Laboratory Animal Use License No. SYXK (Chuan) 2018 − 211], with free access to standard chow and water.

After acclimatization, the mice were randomly assigned to four groups: control (C), exercise (E), tumor-bearing (T), and tumor-bearing plus exercise (TE), with 12 mice in each group. There were no significant differences in body weight among the groups at baseline. The design and reporting of the animal experiments followed the ARRIVE 2.0 guidelines [8].

### Establishment of the tumor-bearing model

CT26 murine colon carcinoma cells (catalog no. CL-0071) were purchased from Procell Life Science & Technology Co., Ltd. (Wuhan, China) and cultured at 37 °C in an atmosphere containing 5% CO₂ using complete medium specifically formulated for CT26 cells. The medium was replaced every 3 days, and cells were passaged at a ratio of 1:3 to 1:5 to maintain logarithmic growth.

On the day of inoculation, cells were digested with 0.25% trypsin-EDTA for approximately 2–3 min, and digestion was terminated with culture medium containing 10% fetal bovine serum (FBS). After centrifugation at 1000 rpm for 5 min, the cells were resuspended in phosphate-buffered saline (PBS) and counted using trypan blue staining. Cell viability was ≥ 98%. A cell suspension was then prepared in sterile PBS at a concentration of 1 × 10⁷ cells/mL.

Mice in the T and TE groups were anesthetized by intraperitoneal injection of sodium pentobarbital at a dose of 50 mg/kg. The dose was calculated according to the individual body weight of each mouse. Adequate anesthesia was confirmed by loss of the pedal withdrawal reflex before inoculation. The mice were then subcutaneously inoculated with 0.2 mL of the cell suspension (2 × 10⁶ cells/mouse) into the left dorsolateral region of the hind limb to establish the tumor-bearing model. Mice in the C and E groups received an equal volume of normal saline at the same site under the same anesthetic procedure. After inoculation, mice were kept under observation until full recovery from anesthesia.

### Aerobic exercise intervention

Formal exercise training was initiated on the day following CT26 inoculation. The formal training protocol consisted of running at 14 m/min for 60 min/day, 6 days/week, for a total of 4 weeks [9]. If an individual mouse failed to complete the target intensity during a given session, the speed in the subsequent session was reduced by approximately 20% and then gradually restored to the target level after recovery [9]. Exercise completion and abnormal responses were recorded during each intervention session, and short rest periods were provided when necessary to ensure animal safety and compliance. To rule out the confounding effects of environmental and handling stress, mice in the non-exercise groups (C and T groups) were placed on a stationary treadmill for 10–15 min daily during the experimental period. A forced treadmill protocol was selected over voluntary wheel running, and resistance training was not chosen, primarily to ensure strict, standardized control over exercise intensity, duration, and frequency across all mice. This approach allows for a highly reproducible aerobic intervention and minimizes inter-individual variability, which is often confounded by the erratic running patterns of voluntary wheel use, consistent with previously reported exercise paradigms [10].

### Sample collection and processing

At the end of the 4-week intervention, mice were euthanized by intraperitoneal overdose of sodium pentobarbital at a dose of 150 mg/kg. The dose was calculated according to the individual body weight of each mouse. Loss of consciousness and deep anesthesia were confirmed by the absence of pedal withdrawal and corneal reflexes. Death was confirmed by the cessation of spontaneous respiration and heartbeat before tissue collection. This euthanasia procedure was selected to minimize pain and distress and to allow rapid collection of tumor and gastrocnemius tissues for subsequent histological and transcriptomic analyses. Tumors and gastrocnemius muscles were then rapidly dissected. Tumor mass and tumor-free body weight (body weight minus tumor mass) were recorded. Part of each gastrocnemius muscle sample was fixed in 4% paraformaldehyde for hematoxylin-eosin (HE) staining. For quantitative assessment of myofiber caliber, myofiber cross-sectional area (CSA) was measured using H&E-stained transverse sections. Images were captured from randomly selected non-overlapping fields under the same magnification. Myofibers with clear boundaries and approximately transverse orientation were included, whereas obliquely sectioned or damaged fibers were excluded. For each mouse, 50 myofibers were measured using ImageJ, and the mean CSA value was calculated and used as a single biological replicate for group-level statistical analysis. All image analyses were conducted in a blinded manner.

### Histomorphological observation of the gastrocnemius muscle

Fixed gastrocnemius muscle samples were subjected to routine tissue dehydration, paraffin embedding, and sectioning. The sections were deparaffinized, rehydrated, stained with hematoxylin, differentiated in hydrochloric acid alcohol, blued, counterstained with eosin, dehydrated through a graded alcohol series, cleared in xylene, and mounted with neutral resin. Morphological changes in the gastrocnemius muscle were observed under a light microscope, and images were acquired [11].

### Total RNA extraction and transcriptome sequencing

Total RNA was extracted using TRIzol reagent (Invitrogen, USA). RNA purity and concentration were measured using a NanoDrop 2000 spectrophotometer (Thermo Scientific, USA), and RNA integrity was assessed using an Agilent 2100 Bioanalyzer (Agilent Technologies, USA). Qualified RNA samples from four randomly selected mice per group ($*n* = 4$ per group) were used to construct sequencing libraries with the VAHTS Universal V5 RNA-seq Library Prep Kit (Vazyme, China). Sequencing was performed by OE Biotech Co., Ltd. (Shanghai, China) on the Illumina NovaSeq 6000 platform using a 150-bp paired-end sequencing strategy.

### Transcriptomic data processing and differential expression analysis

Raw sequencing data were processed with fastp [12]to remove adapter sequences and low-quality reads, yielding clean reads. The clean reads were aligned to the *Mus musculus* reference genome (GRCm39) using HISAT2 [13]. Gene expression counts were generated using HTSeq-count [14], and fragments per kilobase of transcript per million mapped reads (FPKM) values were calculated. Principal component analysis (PCA) and sample clustering were performed in R (v3.2.0) to evaluate biological replicates.

Differentially expressed genes (DEGs) were identified using DESeq2, with thresholds of *q* < 0.05 and fold change > 2 or < 0.5. Hierarchical clustering analysis of DEGs was performed, and heatmaps were generated to visualize expression patterns. Gene Ontology (GO) and Kyoto Encyclopedia of Genes and Genomes (KEGG) enrichment analyses were subsequently conducted using a hypergeometric distribution algorithm, and the results were presented as bubble plots and bar plots. In addition, gene set enrichment analysis (GSEA) was performed using GSEA software to examine the enrichment of predefined gene sets across different experimental groups and to explore the potential molecular pathways through which exercise regulates tumor-associated skeletal muscle atrophy. The fold-change thresholds (FC > 2 or < 0.5) used in DESeq2 were selected to prioritize genes with robust expression changes and reduce false positives. While this strict cutoff enhances specificity, it may bias enrichment analyses toward highly regulated genes and overlook modest but biologically relevant transcriptional changes. Re-analysis of the dataset using a relaxed alternative cutoff (FC > 1.5) demonstrated that while the absolute number of DEGs expanded, the core enriched biological pathways remained highly consistent. Furthermore, because our WGCNA network and module construction were independently based on the top 2000 highly variable genes rather than the DESeq2-derived DEGs, alternative fold-change thresholds exerted no mathematical influence on the subsequent network results.

### Functional enrichment analysis

Differentially expressed genes were imported into the clusterProfiler R package [15] (v4.0.5) for Gene Ontology (GO) enrichment analysis, including biological process (BP), cellular component (CC), and molecular function (MF), as well as KEGG pathway analysis. A threshold of *p* < 0.05 was used to identify significantly enriched functional terms. The results were visualized as bubble plots and bar plots using the ggplot2 R package to further reveal the potential functions and pathway-level roles of the DEGs.

### Weighted gene co-expression network analysis (WGCNA)

To balance network stability and module resolution, the top 2000 genes ranked by expression variance were selected for weighted gene co-expression network analysis (WGCNA). We explicitly note that this top-variance selection may overlook biologically meaningful genes displaying lower expression variance. A weighted co-expression network was constructed and modules were identified using the WGCNA R package [6, 16], and module detection was performed using the dynamic tree cut algorithm [17]. Module-trait correlation analysis and enrichment analysis were subsequently carried out (see Sect.  1.7). Based on the scale-free topology criterion, the soft-thresholding power was set at β = 6 because it achieved a scale-free topology fit index (R²) greater than 0.85, maintaining consistency with recent applied studies [18, 19].

Pearson correlation coefficients and corresponding *p* values between each module and the experimental groups (C, E, T, and TE) were further calculated to identify modules significantly associated with phenotypes. For genes within significantly associated modules, GO and KEGG enrichment analyses were performed to characterize their functional features. For ease of correlation analysis, group variables were binarized as trait labels (e.g., is_T and is_TE) for module-trait association analysis. Module membership (MM) and gene significance (GS) were then integrated to identify key candidate genes and provide potential molecular targets for further investigation.

### Statistical analysis

All data are presented as mean ± SD. Continuous variables were first assessed for normality and homogeneity of variance. For variables measured across the four experimental groups (C, E, T, TE), two-way ANOVA was used to examine the main effects of tumor burden and exercise intervention, as well as their interaction. Post hoc multiple comparisons were performed using Tukey’s test. For variables involving only two groups, such as tumor-related measurements in T and TE groups, independent-samples t tests or Mann–Whitney U tests were used as appropriate. *P* < 0.05 was considered statistically significant.

For data not meeting the assumptions of normality or homogeneity of variance, the Kruskal-Wallis test was used for comparisons among four groups, followed by Dunn’s multiple-comparison correction. The Mann-Whitney U test was used for two-group comparisons. The significance level was set at α = 0.05. Statistical analyses were performed using SPSS 26.0, and figures were generated using GraphPad Prism 9.

## Results

### Tumor formation rate and tumor growth

**Fig. 1:**
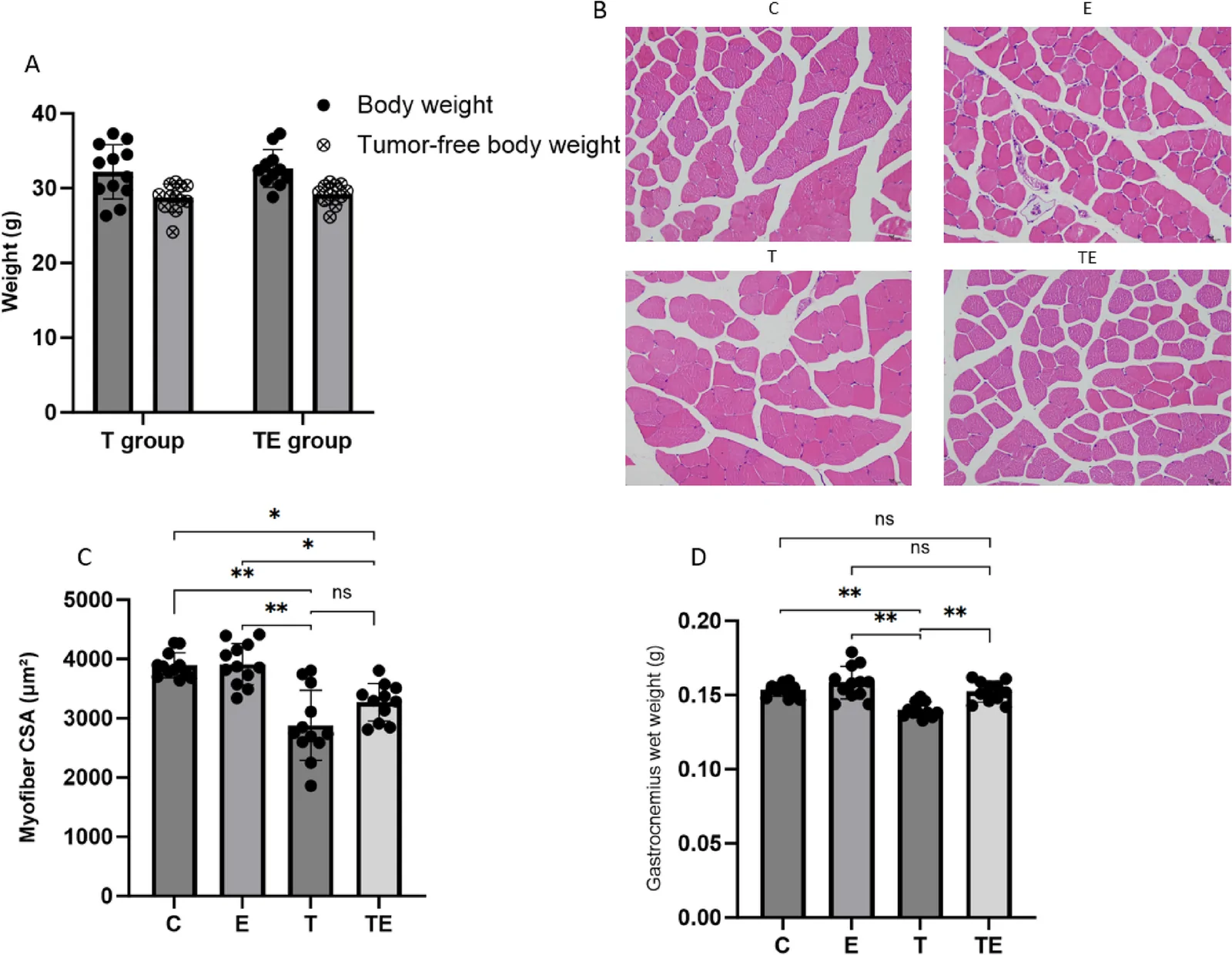
Effects of aerobic exercise on gastrocnemius muscle in CT26 tumor-bearing mice (*n* = 12 mice per group for C, E, and T groups; *n* = 11 mice for TE group). **A** Tumor-free body weight. Each dot represents one mouse; data are mean ± SD. Two-way ANOVA followed by Tukey’s post hoc test was used; no significant differences were observed among groups. **B** Representative H&E-stained images of gastrocnemius muscle. Muscle fibers in C and E groups were well aligned and morphologically intact. T group showed disorganized fibers, widened interstitial spaces, and structural damage. TE group suggested partial attenuation of fiber shrinkage compared with T group. Scale bar = 50 μm. **C** Quantitative analysis of myofiber cross-sectional area (CSA). Each dot represents one mouse; 50 myofibers per mouse were measured for the mean CSA. Data are mean ± SD. Two-way ANOVA with Tukey’s post hoc test was applied. Significant comparisons: C vs. T **; E vs. T **; C vs. TE *; E vs. TE *; T vs. TE ns (**p < 0.01; *p < 0.05; ns = not significant). **D** Gastrocnemius wet weight. Each dot represents one mouse; data are mean ± SD. Two-way ANOVA with Tukey’s post hoc test was applied. Significant comparisons: T vs. C **; T vs. E **; T vs. TE **; C vs. E ns; C vs. TE ns; E vs. TE ns (**p < 0.01; *p < 0.05; ns = not significant)

After subcutaneous inoculation of CT26 cells, all 12 mice in the T group successfully developed tumors, whereas 11 of the 12 mice in the TE group successfully developed tumors, corresponding to tumor formation rates of 100% and 91.7%, respectively. Throughout the experimental period, mice in all groups generally tolerated the experimental procedures. During the observation period, tumor-bearing mice showed a poorer general condition than control mice, as reflected by reduced activity and decreased food intake, and tumor burden showed an increasing trend over time. Compared with the T group, tumor growth-related indices in the TE group showed a downward trend. There was no statistically significant difference in tumor-free body weight among the four groups. Two-way ANOVA showed no significant main effect of tumor burden, exercise intervention, or tumor × exercise interaction (Fig. 1A).

### Histological and quantitative changes in gastrocnemius muscle

HE staining showed that the gastrocnemius muscle fibers in the C and E groups were relatively well aligned and morphologically intact. In contrast, the T group exhibited disorganized muscle fiber arrangement, widened interstitial spaces, and irregular morphology with structural damage in some muscle fibers (Fig. 1B). Compared with the T group, the TE group showed a relatively more orderly arrangement of muscle fibers, suggesting partial attenuation of fiber shrinkage.

Quantitative analysis of myofiber CSA supported the histological observations. The mean CSA values were: C 3893.842 ± 212.635 μm², E 3912.414 ± 349.900 μm², T 2881.936 ± 591.728 μm², TE 3263.697 ± 302.381 μm². Two-way ANOVA indicated a significant main effect of tumor burden, while the main effect of exercise and the tumor × exercise interaction were not significant. Post hoc Tukey comparisons showed significant reductions in T vs. C/E, while the TE group showed a trend toward recovery compared to the T group. Although the TE group showed higher mean CSA than the T group, this difference did not reach statistical significance, indicating that the protective effect of exercise at the fiber level may be modest at this stage (Fig. 1C).

Gastrocnemius wet weight differed among the four groups. Two-way ANOVA revealed significant main effects of tumor burden and exercise intervention, with no significant tumor × exercise interaction. Post hoc Tukey comparisons showed that the T group had significantly lower wet weight than the C and E groups, while the TE group was significantly higher than the T group. The mean ± SD values were: C 0.1534 ± 0.0043 g, E 0.1584 ± 0.0109 g, T 0.1403 ± 0.0052 g, TE 0.1524 ± 0.0067 g (Fig. 1D). These findings suggest that aerobic exercise may partially improve gastrocnemius wet weight and histomorphological abnormalities associated with tumor-bearing conditions.

### Sequencing quality control and sample clustering (QC/PCA)

**Fig. 2:**
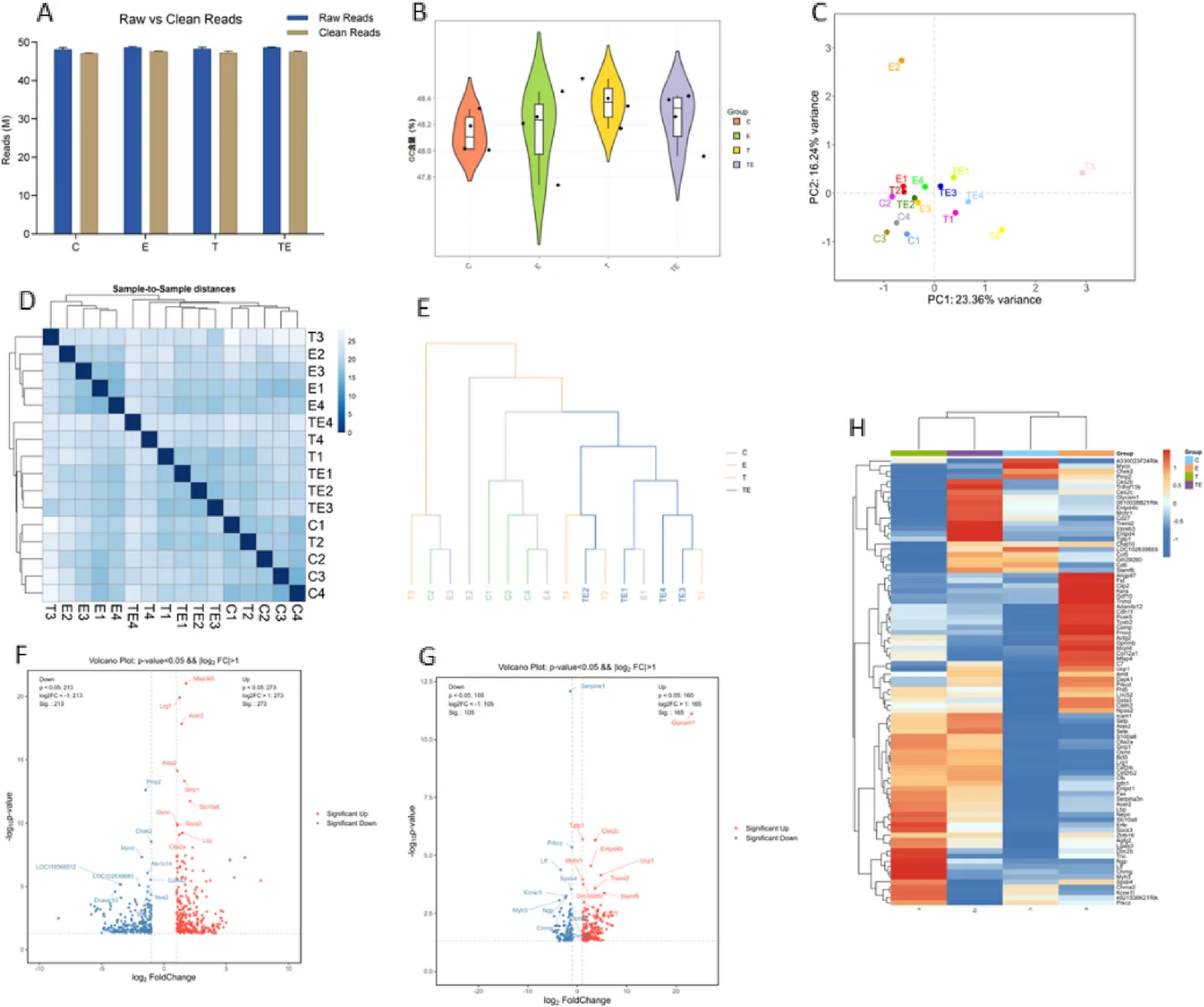
RNA-seq quality control, sample clustering, and differential expression analysis. **A** Comparison of raw reads and clean reads in each group. **B** GC content distribution of sequenced samples. **C** Principal component analysis (PCA) plot. **D** Sample-to-sample distance heatmap. **E** Hierarchical clustering tree of samples. **F** Volcano plot for the T versus C comparison. **G** Volcano plot for the TE versus T comparison. **H** Heatmap of differentially expressed genes. (*n* = 4 mice randomly selected per group for RNA-seq analysis)

The RNA quality of gastrocnemius muscle samples randomly selected from four mice per group (*n* = 4) met the requirements for sequencing. A total of 775.22 million clean reads were generated on the Illumina NovaSeq 6000 platform, and 758.15 million clean reads were retained after quality control with fastp. The proportion of Q30 bases exceeded 96% in all samples (Fig. 2A). The GC content distribution was within a reasonable range (Fig. 2B).

PCA showed that samples from the C and E groups clustered closely, whereas the T and TE groups were clearly separated, indicating that exercise intervention significantly altered the transcriptomic profile of skeletal muscle in tumor-bearing mice. The first principal component mainly distinguished tumor-bearing status, whereas the second principal component distinguished exercise status, which was consistent with the two-factor characteristics of the four-group design (Fig. 2C). The sample clustering heatmap and hierarchical clustering dendrogram further indicated marked intergroup differences and good intragroup reproducibility (Fig. 2D-E).

Differential expression analysis identified 486 differentially expressed genes (DEGs) in the T group compared with the C group, including 273 upregulated and 213 downregulated genes. In the TE group compared with the T group, 270 DEGs were identified, including 165 upregulated and 105 downregulated genes. The volcano plots showed significant differential expression of multiple genes related to muscle atrophy and immune responses among groups (Fig. 2F-G), and the heatmap further indicated distinct expression patterns across groups (Fig. 2H).

### Differential expression profiles and functional enrichment (DEGs, GO/KEGG, and GSEA)

**Fig. 3:**
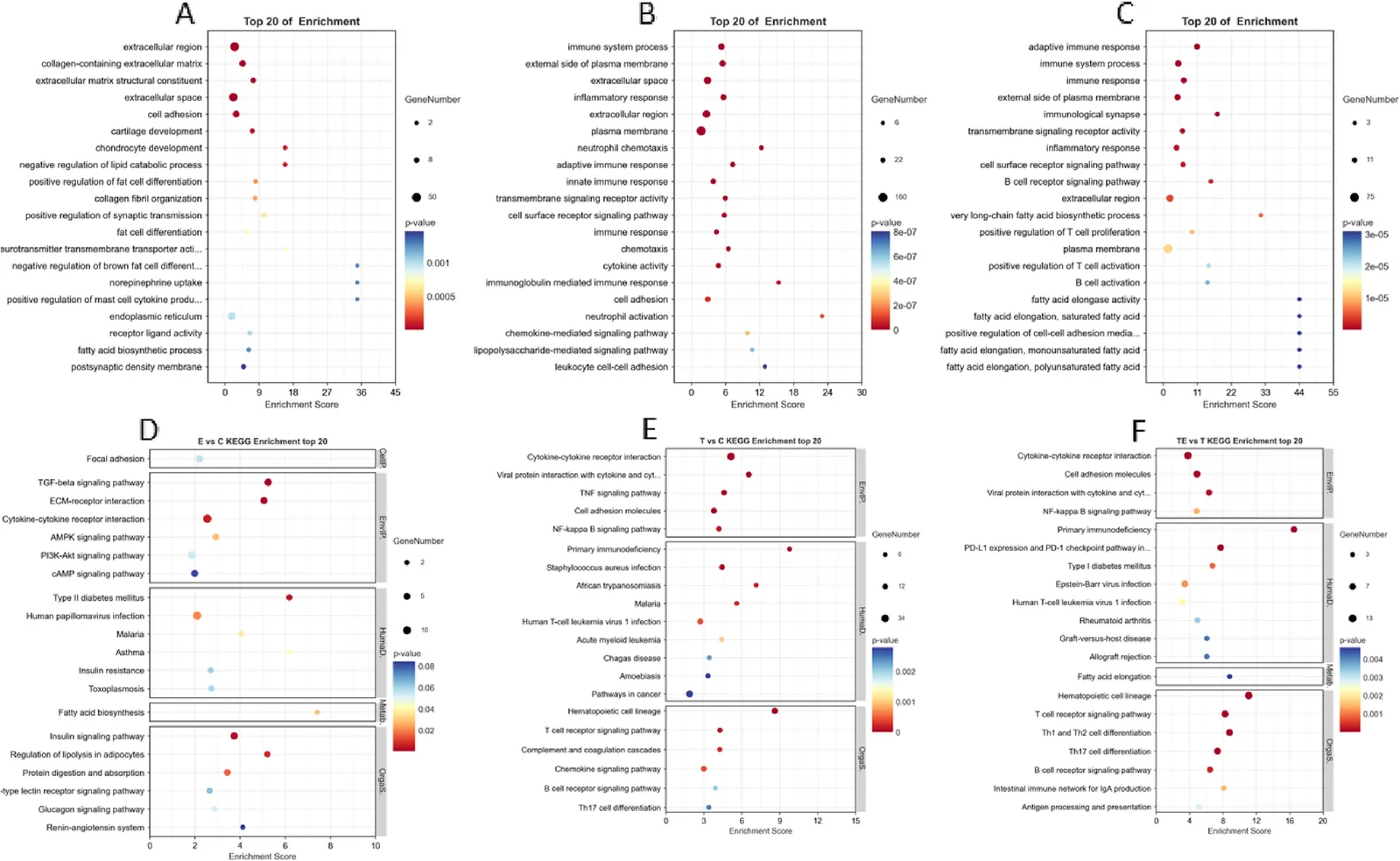
Functional enrichment analysis of differentially expressed genes. **A**–**C** Gene Ontology (GO) enrichment results, including representative terms from the biological process, cellular component, and molecular function categories. **D**–**F** Kyoto Encyclopedia of Genes and Genomes (KEGG) enrichment results showing representative pathways associated with the differential expression profiles. (Analysis based on the same RNA-seq dataset derived from the identical 4 mice per group described in Fig. 2, *n* = 4 per group)

Functional enrichment analysis of DEGs showed that, in the GO analysis, the differentially expressed genes were mainly enriched in biological processes related to muscle contraction, energy metabolism, and cytoskeletal regulation. In terms of cellular components, these genes were primarily associated with the sarcomere, mitochondrial inner membrane, and cytoskeleton-related structures. For molecular functions, significant enrichment was observed in terms such as actin binding and ATPase activity (Fig. 3A-C). KEGG analysis showed that the DEGs were significantly enriched in glycolysis/gluconeogenesis, the AMPK signaling pathway, the PI3K-Akt signaling pathway, and inflammation-related pathways such as the NF-κB signaling pathway (Fig. 3D-F).

**Fig. 4:**
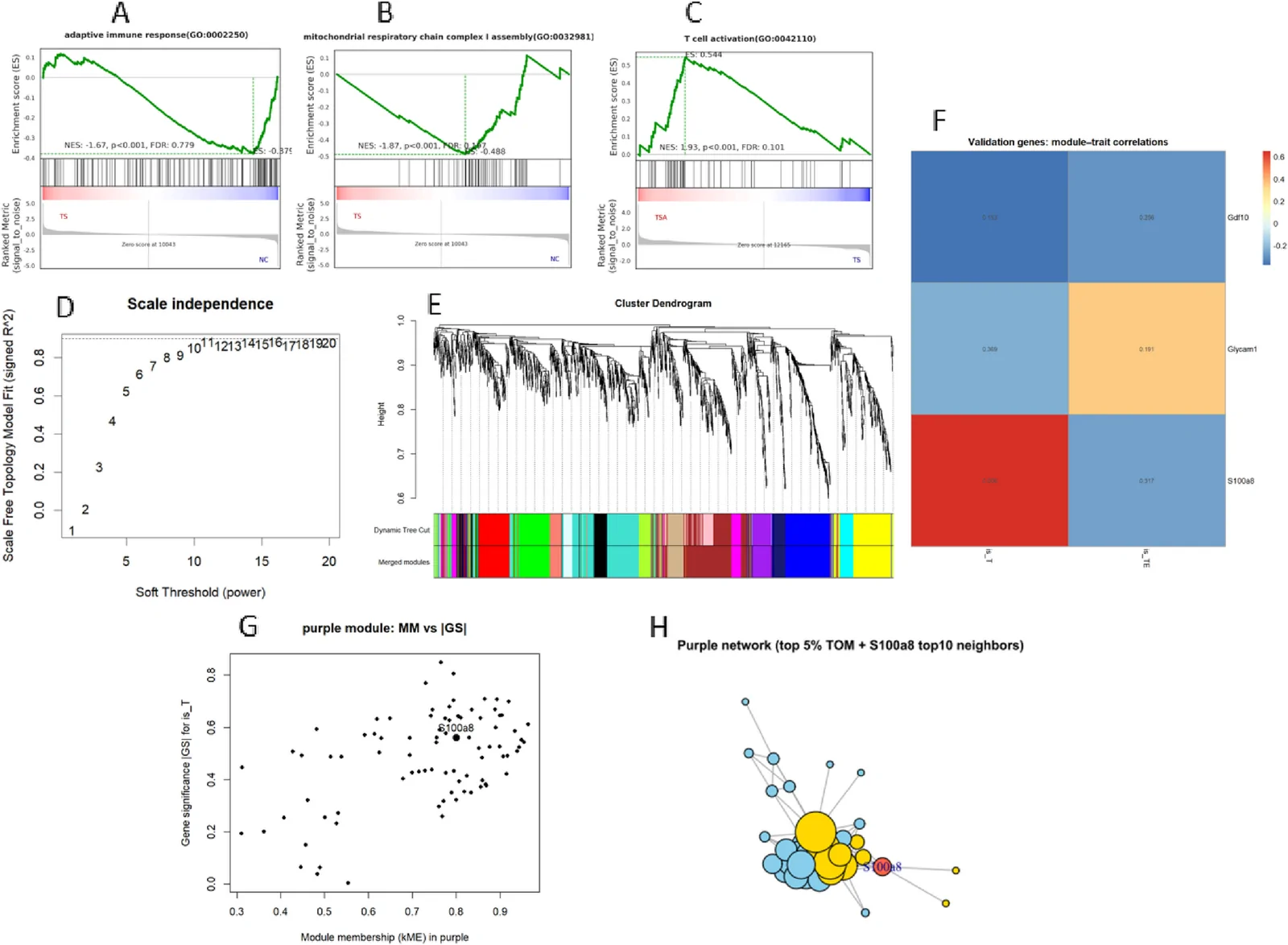
GSEA and WGCNA analyses. **A** GSEA plot for adaptive immune response (GO:0002250) in the T versus C comparison. **B** GSEA plot for mitochondrial respiratory chain complex I assembly (GO:0032981) in the T versus C comparison. **C** GSEA plot for T cell activation (GO:0042110) in the TE versus T comparison. **D** Scale-free topology analysis for soft-threshold selection. **E** Gene dendrogram and module assignment. **F** Module-trait correlation heatmap. **G** Scatter plot of module membership versus gene significance in the purple module. **H** S100a8-centered subnetwork within the purple module. (Analysis based on the same RNA-seq dataset derived from the identical 4 mice per group described in Fig. 2, *n* = 4 per group)

GSEA showed that, in the comparison between the T and C groups, gene sets related to mitochondrial respiratory chain complex I assembly (GO:0032981) and adaptive immune response (GO:0002250) were significantly downregulated. In the comparison between the TE and T groups, the gene set related to T cell activation (GO:0042110) was significantly upregulated (Fig. 4A-C). These results suggest that skeletal muscle in the tumor-bearing state exhibits marked inflammation-metabolism imbalance, and that aerobic exercise may improve this abnormal state by remodeling relevant transcriptional programs.

### Co-expression modules and key genes identified by WGCNA

A weighted gene co-expression network was constructed based on the top 2000 highly variable genes. At the soft-thresholding power of β = 6, the network satisfied the scale-free topology criterion while maintaining reasonable connectivity (Fig. 4D). Hierarchical clustering based on topological overlap matrix (TOM) distance, followed by module merging, identified a total of eight stable modules (Fig. 4E).

Module-trait relationship analysis showed that the purple module was significantly positively correlated with is_T (*r* ≈ 0.652, *p* ≈ 0.006), whereas the greenyellow module was significantly negatively correlated with is_TE (*r* ≈ -0.520, *p* ≈ 0.039) (Fig. 4F). Within the purple module, module membership (MM) was positively correlated with gene significance (|GS|), and S100a8 showed relatively high MM and |GS| values, suggesting that it exhibited the characteristics of a candidate hub gene in this module (Fig. 4G). To improve data transparency, the normalized expression values (FPKM) of S100a8 across all four experimental groups are provided in Supplementary Table S1. Further comparison showed that the module containing S100a8 was strongly associated with the tumor-bearing phenotype, whereas Gdf10 and Glycam1 did not receive statistical support. To visualize the network structure, the top 5% of strong TOM-based edges were retained and supplemented with the top 10 neighbors of S100a8, which was clearly highlighted in the subnetwork (Fig. 4H).

Taken together, the exercise-related molecular changes observed in this study mainly pointed to the inflammation-related purple module and its representative gene S100a8, whereas Gdf10 and Glycam1 were not supported statistically, suggesting that their roles still need to be further validated in larger samples.

## Discussion

Cancer-associated skeletal muscle atrophy is one of the common complications of malignant tumors, and its development is closely associated with persistent inflammatory activation, enhanced protein degradation, and impaired energy metabolism [1, 3, 4, 20]. Identifying broadly applicable intervention strategies and their molecular basis is of great importance for improving patients’ quality of life and tolerance to treatment [3, 20]. In the present study, using a CT26 tumor-bearing mouse model, we combined phenotypic assessment of gastrocnemius muscle with transcriptomic analysis and weighted gene co-expression network analysis (WGCNA) to explore transcriptomic alterations associated with tumor-related muscle wasting and aerobic exercise intervention.

Our results showed that the tumor-bearing state was associated with reduced gastrocnemius wet weight and disorganized muscle fiber arrangement with structural abnormalities, reflecting phenotypic features of tumor-associated muscle wasting. Compared with the T group, the TE group exhibited partial improvements in gastrocnemius wet weight and a trend toward histomorphological recovery following aerobic exercise intervention, whereas the difference in tumor-free body weight did not reach statistical significance. These findings suggest that the observed effect of exercise was more evident at the local gastrocnemius level than at the whole-body weight level. Importantly, our quantitative assessment of myofiber CSA substantiated the occurrence of authentic tumor-induced muscle atrophy, providing a more direct, cell-intrinsic structural correlate that effectively decouples true muscle fiber shrinkage from confounding factors such as tissue edema or inflammatory infiltration. This discrepancy further addresses whether the modeled phenotype reflects a localized, muscle-specific wasting rather than systemic, whole-body cachexia. Given that total tumor-free body weight remained statistically unchanged despite marked gastrocnemius structural damage and mass loss, our 4-week CT26 model likely captures an early, muscle-specific phase of wasting. During this initial stage, high-metabolic locomotor muscles like the gastrocnemius serve as primary, sensitive targets for tumor-induced disruption before severe, multi-organ systemic cachexia fully manifests. This study should therefore be interpreted within the context of an early-stage, pre-cachectic model, rather than advanced systemic cachexia. These findings provided a phenotypic basis for subsequent transcriptomic and network analyses. These observations are generally consistent with previous studies suggesting that aerobic exercise can attenuate muscle wasting-related phenotypes in tumor-bearing animals [2, 7, 21].

Transcriptomic and enrichment analyses indicated that tumor burden triggered a marked inflammation-metabolism imbalance within the skeletal muscle, characterized by the upregulation of proteolytic programs and the concomitant downregulation of pathways governing energy supply and structural maintenance [20, 22]. Our findings demonstrate that aerobic exercise partially reversed these transcriptional aberrations, suggesting a coordinated remodeling of local molecular networks rather than a uniform suppression of immune activity [7, 21]. Mechanistically, this exercise-induced protection can be interpreted through two alternative, non-mutually exclusive frameworks: systemic versus muscle-intrinsic effects. Systemically, regular aerobic exercise is well-documented to mitigate circulating cachexigenic cytokines and improve whole-body metabolic homeostasis [2, 7]. Intrinsically, repeated mechanical loading and metabolic stress from muscle contraction directly activate local energetic sensors (such as the AMPK and PI3K-Akt pathways) and structural stabilization programs within the myofibers [2, 4]. Given that our transcriptomic data revealed a localized restoration of metabolic and structural transcripts in the gastrocnemius, these alterations provide preliminary, transcript-level indications that muscle-intrinsic pathways may participate in counteracting tumor-induced wasting in this early stage, even though systemic anti-inflammatory contributions cannot be ruled out.

WGCNA further revealed that the inflammation-related module closely associated with the tumor-bearing phenotype had relatively high explanatory value in the present study. Within the purple module, S100a8 showed relatively high module membership (MM) and gene significance (GS), indicating that it may serve as a candidate hub gene. Previous studies have suggested that S100A8/A9 can participate in inflammation amplification, immune cell recruitment, and metabolic dysregulation, and may be involved in tumor-associated cachexia. In the context of the present study, S100a8 may represent a candidate node associated with inflammatory and metabolic alterations, but its causal role requires further functional validation. It should be noted, however, that the current evidence is derived mainly from transcriptomic and co-expression network-based association analyses and is insufficient to establish a causal role. Further validation at the mRNA and protein levels, together with tissue localization and functional intervention experiments, is still required to clarify the biological role of S100a8 in the protective effects of exercise.

Several limitations should be acknowledged. First, although gastrocnemius wet weight and H&E staining provided phenotypic evidence of muscle wasting–related changes, no functional assessments of muscle strength or endurance were performed, partly due to experimental equipment constraints and our primary focus on early-stage transcriptional network remodeling. Consequently, it remains unclear whether the observed changes in gastrocnemius wet weight and histological features translated into functional recovery. Second, key hub genes identified through WGCNA were not independently validated at the mRNA or protein level (e.g., via qRT-PCR, Western blot, or immunohistochemistry). In addition, specific biochemical markers related to protein synthesis and degradation, as well as their upstream signaling pathways (such as the Akt/mTOR or AMPK cascades), were not experimentally examined. Therefore, these pathway alterations currently represent transcript-level associations rather than confirmed phenotypic adaptations, which warrants cautious translational interpretation. Third, the relatively small RNA-seq sample size (*n* = 4 per group) may limit statistical power and the stability of network inference. Additionally, WGCNA captures correlation-based modular structure and does not account for directionality or causality in gene interactions. Fourth, bulk RNA-seq cannot distinguish transcriptional changes occurring in muscle fibers from those arising in infiltrating immune or stromal cells. Finally, the candidate hub gene S100a8 requires further mechanistic and functional studies to confirm its regulatory role in cancer-associated muscle remodeling. Collectively, these limitations restrict the translational interpretation of our findings, particularly because muscle function is clinically more relevant than muscle mass alone. Future studies incorporating functional assessments, mechanistic validation, and larger sample sizes will be necessary to further clarify the role of exercise in cancer cachexia–associated muscle remodeling.

## Conclusions

Aerobic exercise was associated with a significant improvement in gastrocnemius wet weight and a mitigating trend in histomorphological abnormalities in CT26 tumor-bearing mice, accompanied by changes in inflammation- and metabolism-related transcriptional networks. WGCNA identified S100a8 as a candidate inflammation-related hub gene associated with the tumor-bearing phenotype and exercise intervention, although these findings should be interpreted as transcriptomic and network-based associations rather than causal evidence. Further studies incorporating muscle function tests, protein-level validation, and functional intervention experiments are warranted.

## Supplementary Information

Supplementary Material 1.

Supplementary Material 2.

## Data Availability

The RNA-seq datasets generated and analyzed during the current study have been deposited in the Gene Expression Omnibus (GEO) repository under accession number GSE328094. Other data supporting the findings of this study are available from the corresponding author on reasonable request.

## Abbreviations

AMPK: AMP-activated protein kinase
BP: Biological process
CC: Cellular component
DEGs: Differentially expressed genes
FBS: Fetal bovine serum
FPKM: Fragments per kilobase of transcript per million mapped reads
GC: Guanine-cytosine
GO: Gene Ontology
GSEA: Gene set enrichment analysis
GS: Gene significance
HE: Hematoxylin-eosin
KEGG: Kyoto Encyclopedia of Genes and Genomes
MF: Molecular function
MM: Module membership
PCA: Principal component analysis
PBS: Phosphate-buffered saline
RNA: Ribonucleic acid
SPF: Specific pathogen-free
TOM: Topological overlap matrix
WGCNA: Weighted gene co-expression network analysis
