# Supplementary material for: Aerobic exercise is associated with alleviation of skeletal muscle atrophy in CT26 tumor-bearing mice: insights from transcriptomic and weighted gene co-expression network analyses
Source: BMC Musculoskelet Disord. 2026 Jun 26;27:780. doi: 10.1186/s12891-026-10131-5 (PMC13591731; doi:10.1186/s12891-026-10131-5)
Supplement: Supplementary file 1 [file 12891_2026_10131_MOESM1_ESM.pdf]

**Supplementary Figure S1. High-resolution enlarged individual panels for Figure 4.**

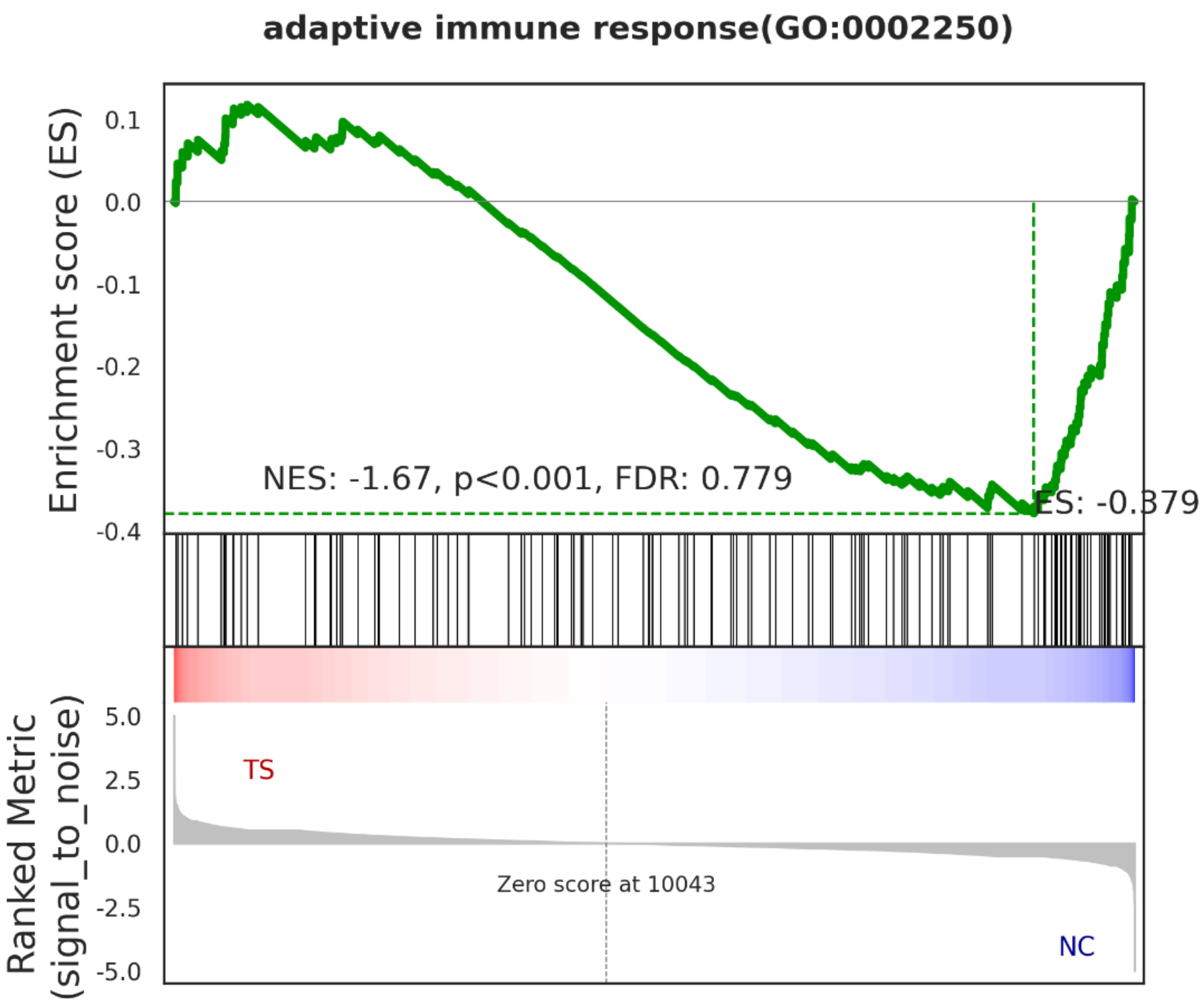


**Supplementary Figure S1A.** GSEA plot for adaptive immune response (GO:0002250) in the T versus C comparison.

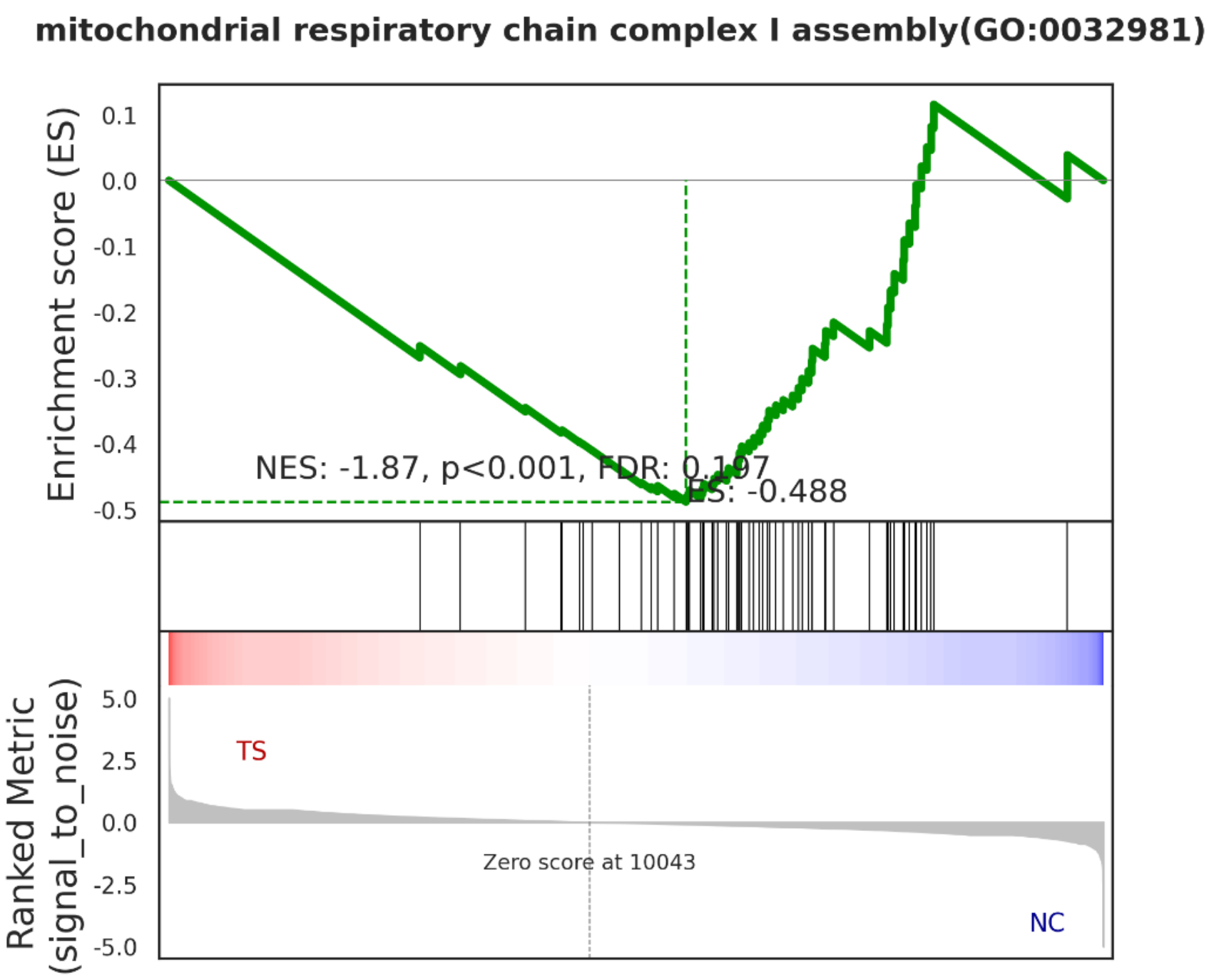


**Supplementary Figure S1B.** GSEA plot for mitochondrial respiratory chain complex I assembly (GO:0032981) in the T versus C comparison.

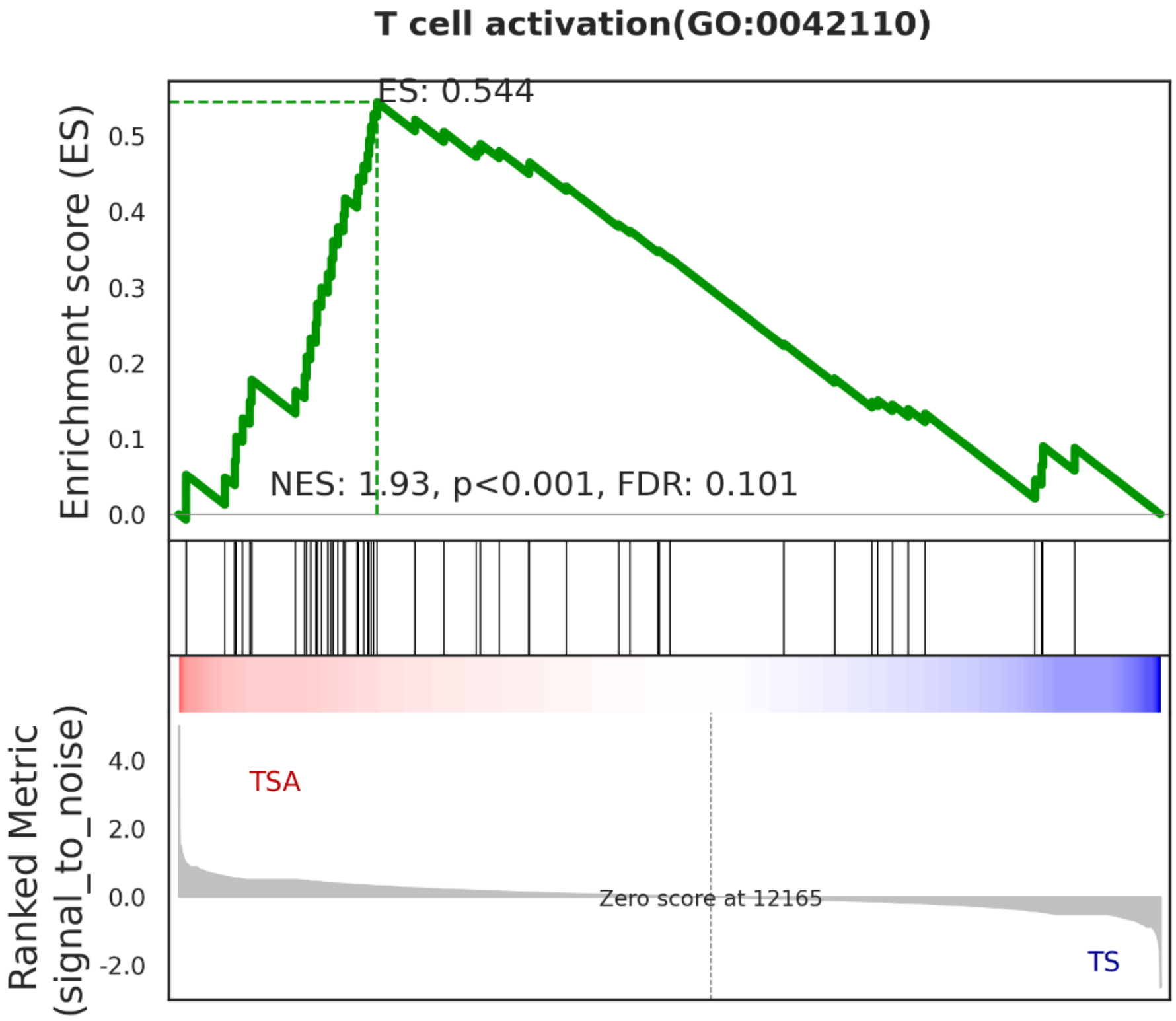


**Supplementary Figure S1C.** GSEA plot for T cell activation (GO:0042110) in the TE versus T comparison.

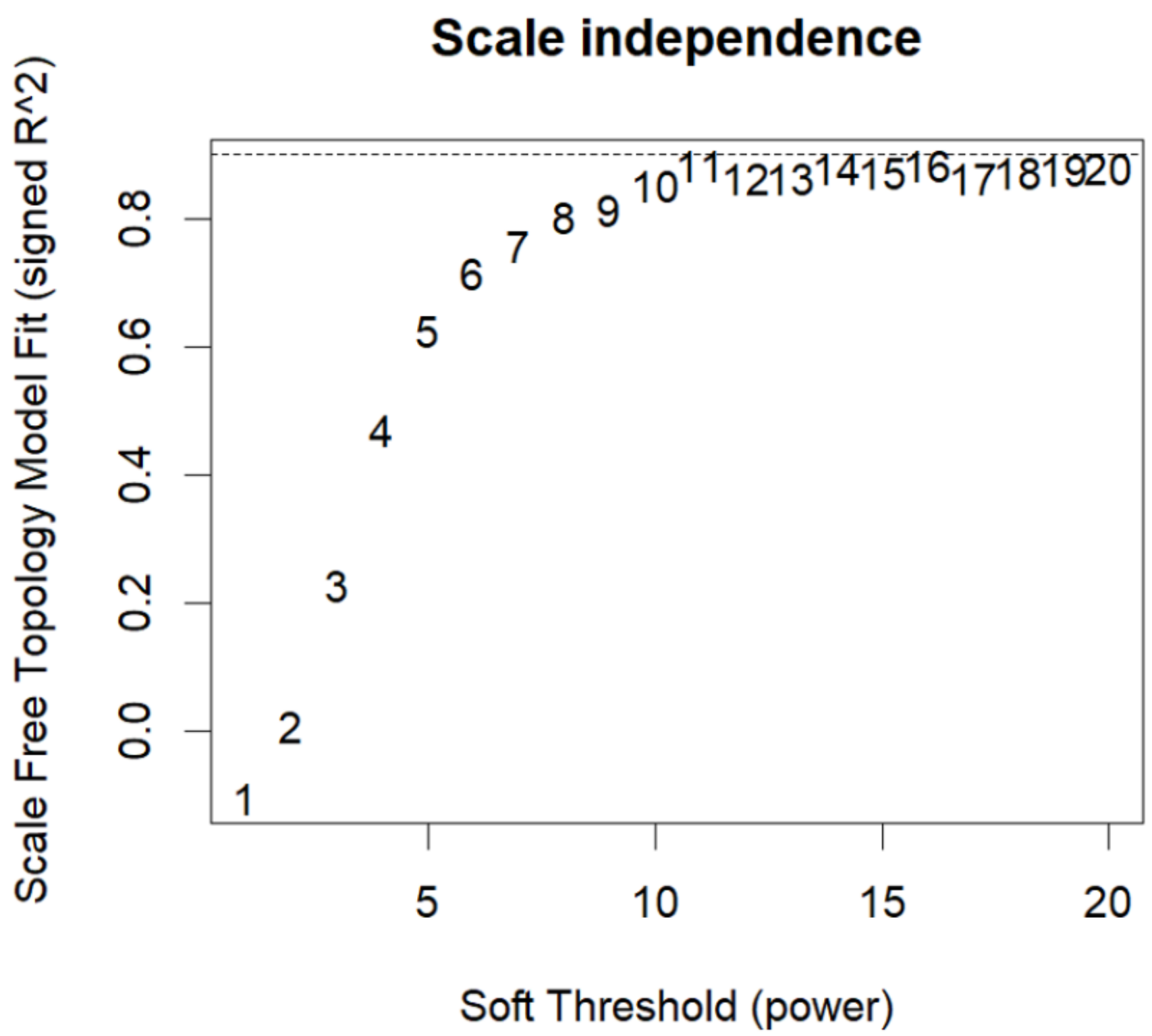


**Supplementary Figure S1D.** Scale-free topology analysis for soft-threshold selection.

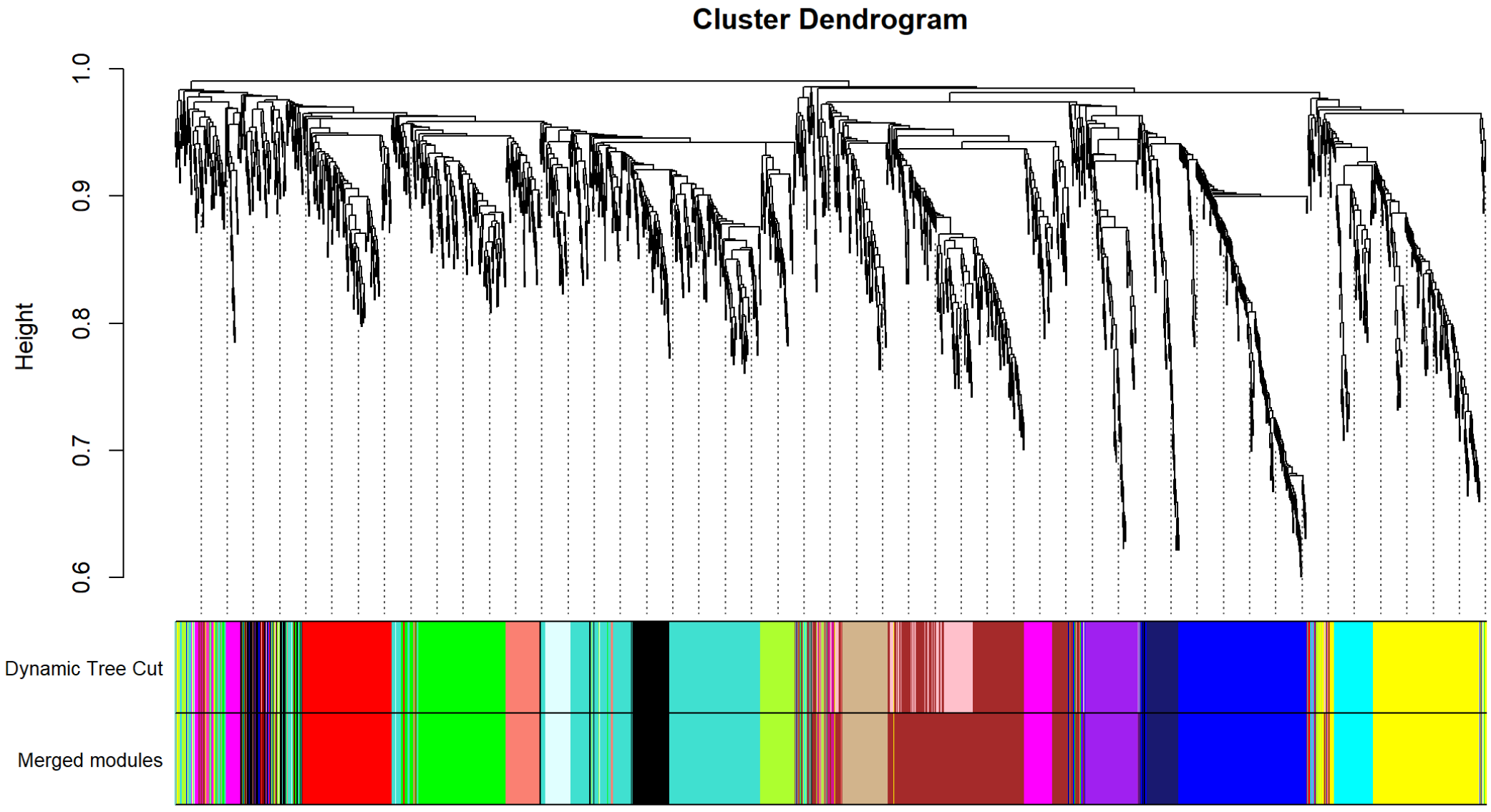


**Supplementary Figure S1E.** Gene dendrogram and module assignment (Cluster Dendrogram).

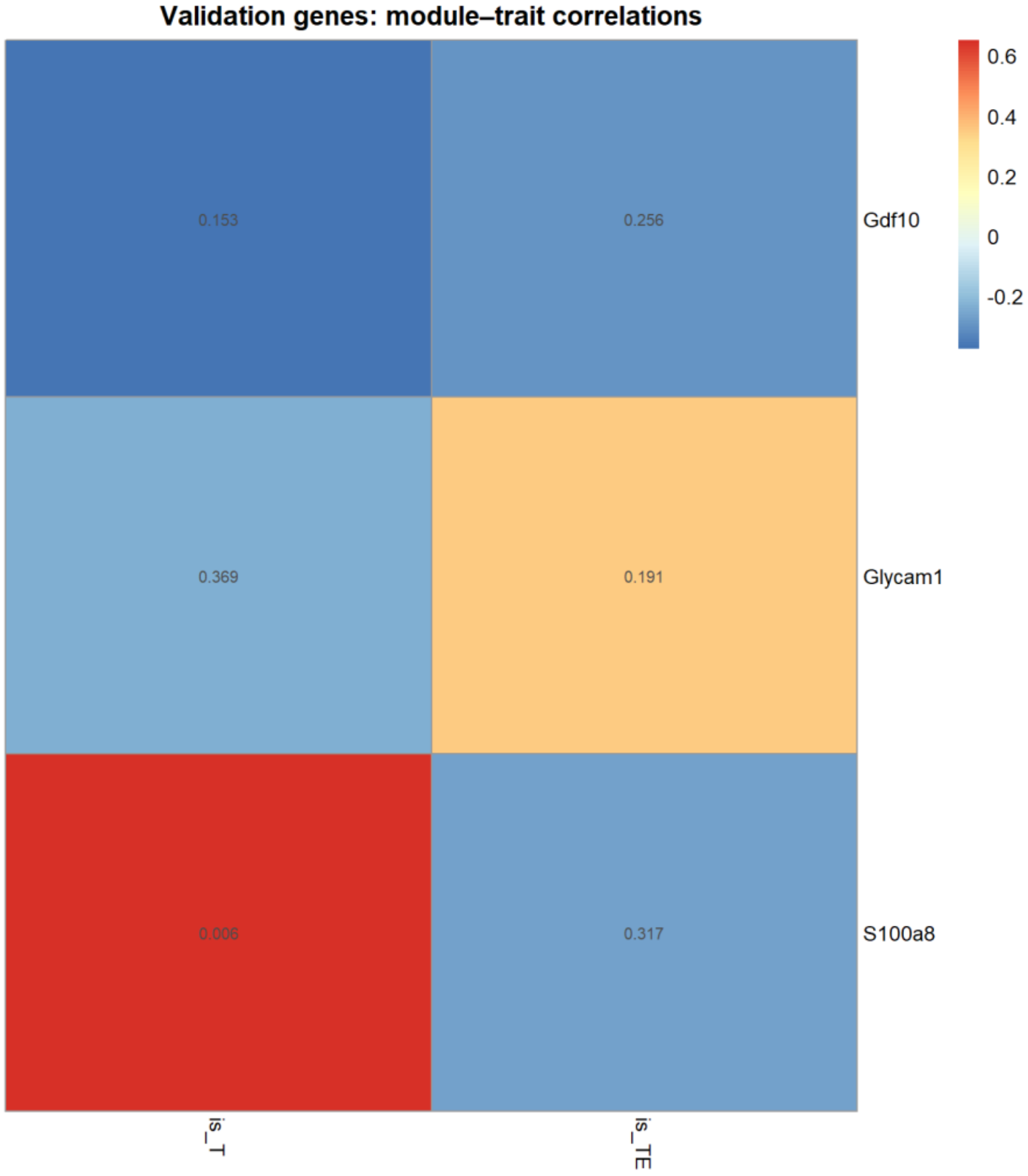


**Supplementary Figure S1F.** Module-trait correlation heatmap.

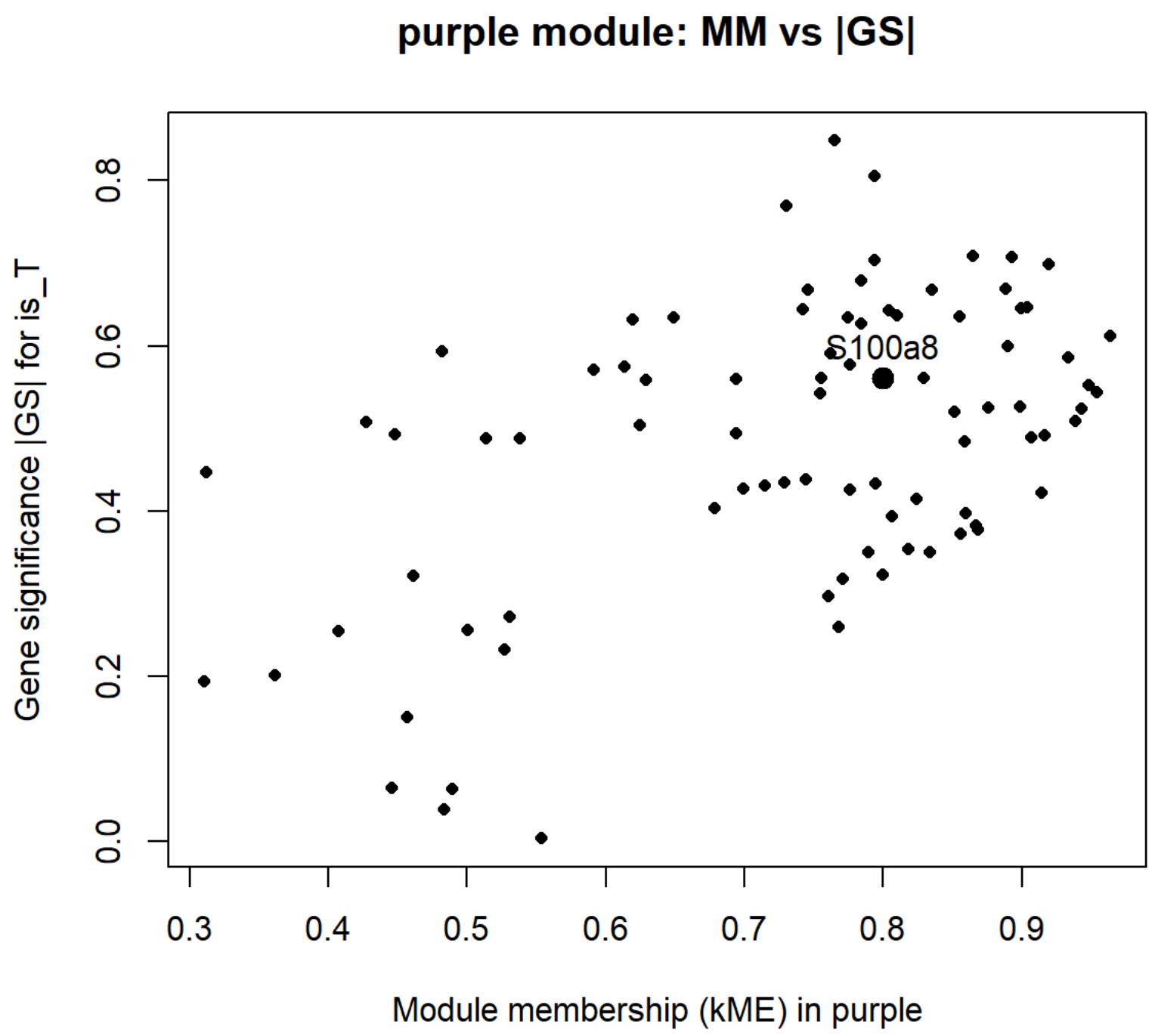


**Supplementary Figure S1G.** Scatter plot of module membership versus gene significance in the purple module.

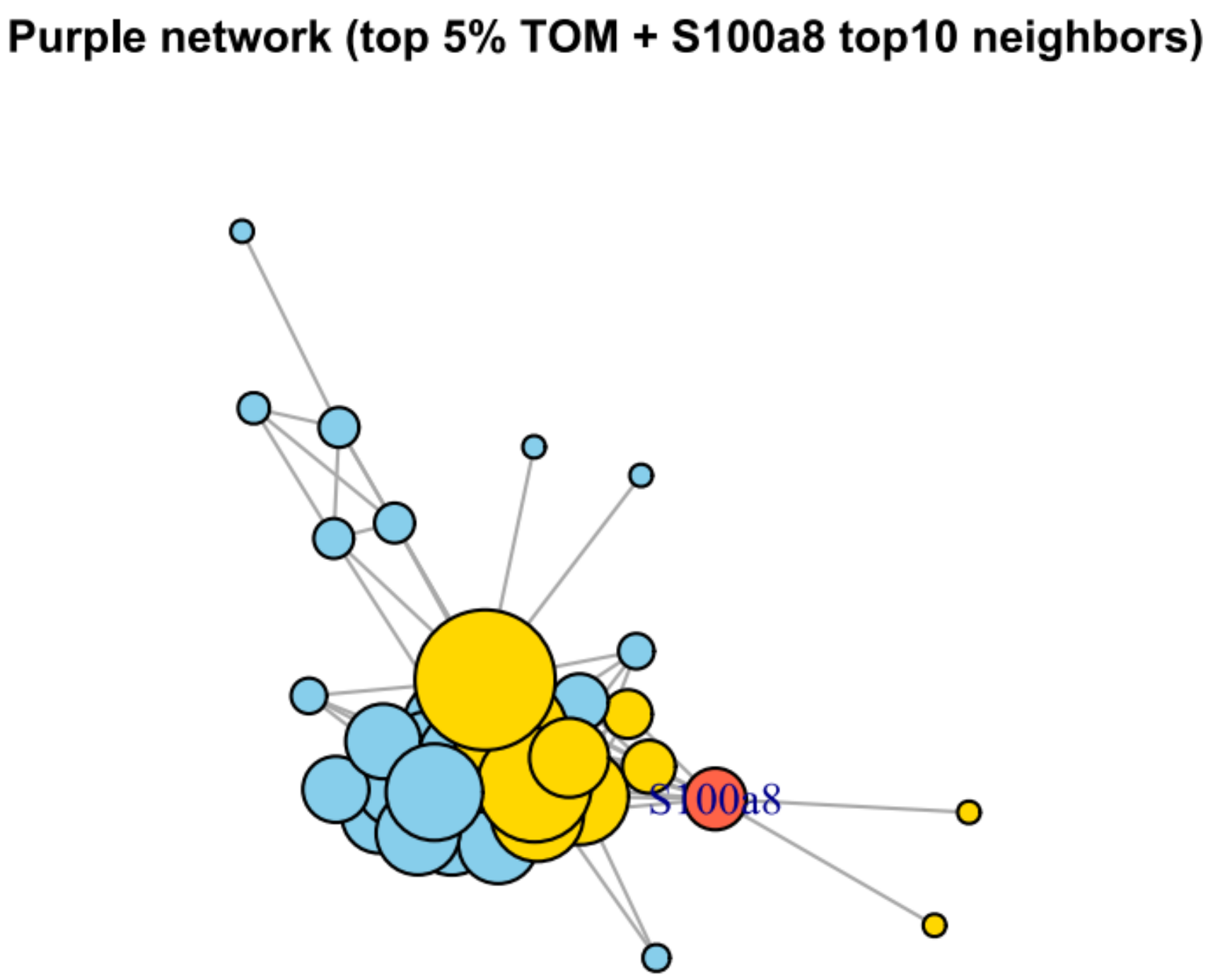


**Supplementary Figure S1H.** *S100a8*-centered co-expression subnetwork within the purple module.
